# Molecular detection of human rhinoviruses in respiratory samples: a comparison of Taqman probe-, SYBR green I- and BOXTO-based real-time PCR assays

**DOI:** 10.1186/1743-422X-11-31

**Published:** 2014-02-18

**Authors:** Julien Dupouey, Laetitia Ninove, Vanessa Ferrier, Odile Py, Céline Gazin, Laurence Thirion-Perrier, Xavier de Lamballerie

**Affiliations:** 1 IRD French Institute of Research for Development, EHESP French School of Public Health, Aix Marseille Univ, UMR_D 190 “Emergence des Pathologies Virales”, Marseille 13005, France; 2 Pôle des Maladies Infectieuses et tropicales Clinique et Biologique, Fédération de Bactériologie-Hygiène-Virologie, Centre Hospitalo-Universitaire Timone, 264 rue Saint-Pierre 13385, Marseille CEDEX 05, France

**Keywords:** BOXTO, SYBR green, Taqman probe, Picornaviridae, Human rhinovirus, Respiratory infections, Molecular diagnosis

## Abstract

**Background:**

Human Rhinoviruses (HRV) are major causative agents of acute respiratory tract infections in all age group and important contributing factors of childhood morbidity and mortality. Clinical presentation is poorly specific and the great antigenic and genetic variability of HRVs renders the biological diagnosis complex. Here, we have evaluated several molecular diagnostic protocols, including Taqman probe-based and intercalating agent-based RT-PCR assays.

**Methods:**

5,627 respiratory samples sent to the laboratory of Virology of the University Hospitals of Marseille, France, from March 2011 to February 2012, were tested using a real-time RT-PCR assay in the 5’NCR of the rhinoviral genome that associated a Taqman probe and the detection of DNA-BOXTO-dye complexes. A sample of 500 BOXTO-positive samples were further tested using the same probe assay (without BOXTO), and a SYBR Green assay (using the same amplification primers). The specific amplification of HRV sequences was assessed by NGS amplicon sequencing.

**Results:**

The Taqman probe RT-PCR assay identified 696/5,627 samples (12,4%) as HRV-positive. BOXTO-positive samples included all probe-positive samples and 1,913 additional samples, of which only 24.3% were confirmed by sequencing. The SYBR Green assay was more specific (16/550 samples were probe-negative/SYBR Green-positive, all confirmed by 5′NCR sequencing), but 3/500 samples were probe-positive/SYBR Green-negative.

**Conclusions:**

Our results highlight the difficulty in detecting HRVs in clinical samples using a single molecular detection system. Amongst the 3 systems tested, the best compromise was obtained with the SYBR Green assay, which, by comparison with our probe-based assay provided an improved sensitivity without altering the detection specificity. Interestingly, a majority of probe-negative/BOXTO- or SYBR Green-positive samples were not associated with mutations in the sequence targeted by the probe. Sequence-based modifications of the secondary structure of the HRV 5′NCR may be associated with a limited access to the probe hybridisation region. Further investigations may identify a test combining a probe based- and an intercalating agent-based detection, which will significantly improve the diagnosis of HRV infections.

## Background

Acute respiratory tract infections (ARIs) represent a major public health problem worldwide, a leading cause of human acute illnesses and an important contributing factor of childhood morbidity and mortality, especially in children under 5 years [1-3]. A majority of ARIs in children have a viral etiology, probably due to absent or incomplete immune protection, sustained viral shedding and high transmissibility amongst hosts [4]. Amongst a variety of RNA and DNA viruses that can infect the respiratory tract, Human Rhinoviruses (*Picornaviridae* family) [5], are recognized as the most prevalent in all age group worldwide [6]. Together with coronaviruses, they represent common causative agents of upper respiratory tract (URT) infections, traditionally defined as common cold [6,7], and thus a major cause of school and work absenteeism since children experience 8–12 and adults 2–3 URT episodes per year, on average [8]. Children are the major reservoir for HRV [9]. The mean age at the first symptomatic HRV infection is 4–6 months (*vs* >6 months for other viruses such as RSV [10]; more than 90% of children have experienced at least one HRV infection by the age of 2 years [11]. HRV infection is most often associated with a non-specific, self-limiting illness with clinical manifestations ranging from an asymptomatic presentation to fever, rhinorrhea, cough and wheezing. However, HRV infections are increasingly involved in otitis media, pneumonia, or chronic obstructive pulmonary disease, especially in infants, elderly and immunocompromised patients [12,13]. In addition, HRV represent a major viral aetiology of asthma exacerbations, with the highest incidence of all respiratory viruses in adults and children >2 years of age (60–65% of viral exacerbations) [14,15]. Although suspected in some studies, there is at the present day no proof of association between clinical severity and HRV species [16-20]. Altogether, these data highlight the predominant role of HRV as a respiratory pathogen especially in early life.

HRVs are small non-enveloped viruses with a single-stranded RNA genome of positive polarity; originally classified in the *Rhinovirus* genus, they have been integrated into the *Enterovirus* genus. Rhinoviruses share with Enteroviruses an identical genomic organization and have similar functional RNA secondary structures, but differ in their acid tolerance, receptor usage, and cell tropism [21]. The genome is approximately 7.2 kb long, and is composed of a 5′non-coding region (5′NCR), followed by a long open reading frame coding for four structural icosahedral capsid proteins (VP4, VP2, VP3 and VP1), and seven non-structural proteins 2A, 2B, 2C, 3A, 3B, 3C and 3D, and terminated by a short 3′UTR and poly A tract. HRVs are highly heterogeneous genetically and antigenically [22,23]. More than 140 serotypes have been described, and these fall into three species, HRV species A (HRV-A; 74 serotypes), HRV-B (25 serotypes) and a novel genetically distinct third genotype HRV-C, comprising 49 designated serotypes recognized in 2006 [24-27].

HRV infections occur throughout the year [28], usually with peaks in spring and autumn in temperate countries [29-31], the prevalence varying from 10% to 60% depending on the population or the period studied. Molecular studies suggest almost equal prevalence of HRVA and HRVC, with a under-representation of HRVB species [11,19,32-39]. A remarkably wide genetic diversity of HRV serotypes can be observed all year long [40].

The diagnosis of HRV infections is important for epidemiological purposes but also for optimising the medical management of patients (*e.g.*, the opportunity for an antibiotic treatment). As clinical presentation is non-specific, it devolves to the diagnostic laboratory to confirm the presence of HRVs [41]. Detection of HRV by culture is slow and complex for HRVA and HRVB, whilst HRVC has been unculturable *in vitro* to date [23]. Serologic diagnosis is virtually impossible due to the number of serotypes, and rapid antigen test kits are not available [4]. Molecular methods such as real-time RT-PCR appear to be the most suitable method, combining short analysis time, high sensitivity, semi-quantification of viral load and the detection of the majority of respiratory viruses with multiplex methods [42-44]. Most of the published systems target the 5′NCR. However, the high genetic diversity of HRVs makes the detection of all variants difficult as evidenced by the alignment of available GenBank 5′NCR sequences. Indeed, a comparison of published HRV-specific PCR primers pairs showed that no single pair could detect all HRVs [45] and that more than one PCR is required for accurate description of HRV epidemiology.

The range of detection of HRV variants by a probe-based real-time PCR assay is predicted to be additionally restricted by mutations in the region of the probe. Accordingly, we have compared the performances of 3 tests for accurately detecting HRV in clinical samples: (i) a probe-based Taqman RT-PCR assay routinely used in our hospital laboratory; (ii) the same test used without the probe and in the presence of SYBR Green I, making theoretically possible to diagnose HRVs amplified by the primers but not detected by the probe; (iii) the same test used with both the probe and in the presence of the BOXTO intercalating agent, making theoretically possible to detect HRV detected and undetected by the probe in the same reaction.

## Methods

### Clinical Samples

5,627 samples (nasopharyngeal aspirates, swabs or saliva specimens) sent to the Laboratory of Virology, University Hospital La Timone (Marseille, France) from March 2011 to February 2012 and stored at -80°C upon reception were studied. They were predominantly from hospital inpatients or patients in emergency departments, who presented with respiratory symptoms.

All samples were analysed using real-time RT-PCR assay using an association of a specific-probe and BOXTO. 500 samples randomly chosen amongst those with a positive BOXTO result (see below) were secondarily tested using (i) the same specific-probe without BOXTO, and (ii) a SYBR Green assay.

### RNA extraction

Each sample (200 μL) was spiked by a mix of T4 and MS2 phages used as internal controls [46]. Nucleic acids extraction was performed using the EZ1 Virus Mini Kit v2.0 and the EZ1 Advanced XL Biorobot (both from Qiagen). The final elution volume was 90 μL.

### HRV molecular diagnosis

i) Taqman probe assay: a real-time one-step RT-PCR reaction was performed using the iScriptTM One-Step RT-PCR Kit (Biorad) and the Biorad CFX96 Real time System thermocycler. The 30 μL final reaction volume contained 10 μL of viral RNA, 0.3 μM of each primer (forward 1: WGCCYGCGTGGCKGCC, forward 2: AGCCYGCGTGGTGCCC; reverse: GAAACACGGACACCCAAAGTAGT) and 0.1 μM of specific probe (6FAM-CTCCGGCCCCTGAATGYGGCTAA-TAMRA); thermal cycling was: reverse transcription at 50°C for 10 min, initial denaturation at 95°C for 5 min, followed by 40 cycles of [95°C for 15 s, 60°C for 30 s]. This hospital diagnostic assay used a standardised hybridisation temperature and primers and probes concentrations were optimised using a classical matrix experimental procedure.

ii) BOXTO: the optimal concentration of BOXTO for was determined according to the manufacurer’s recommendation (TATAA Biocenter). In the case of the Taqman probe assay presented above, the best results were obtained with a 2.5 μM concentration. The protocol adapted from Lind et al. [47] was identical to that of the Taqman probe assay, with the addition of a final melting curve (65°C to 95°C, with an increase of 0.5°C every 5 s), visualised in the “HEX” reading canal.

iii) SYBR Green: the SYBR Green assay was conducted using the QuantiTect SYBR® Green RT-PCR Kit and comparable reaction parameters; thermal cycling was: reverse transcription at 50°C for 30 min, initial denaturation at 95°C for 15 min, followed by 40 cycles of [95°C for 15 s, 60°C for 30 s and 72°C for 30 s], and a final step with a melting visualised in the “HEX” reading canal.

### 5′NCR sequencing

5′NCR PCR products were purified (QIAquick Gel Extraction, QIAGEN). The 215 bp DNA products were tagged and analysed using the Ion Torrent NGS technology without any further fragmentation. Sequencing data were analysed using to the CLC Genomics Workbench bioinformatic software [48].

### Structural analysis of Taqman probe

The on-line software M-fold v3.2 was used to predict the secondary structure of the Taqman probe sequence [49].

### Statistical analysis

Statistical analysis was performed using the IBM SPSS Statistics software in this study.

## Results

Between March 2011 and February 2012, 5,627 respiratory samples were received for detection of respiratory virus infections and analysed in this study.

i) Taqman probe/BOXTO assay:

Using the assay that combined a probe detection and a BOXTO detection:

**Figure 1:**
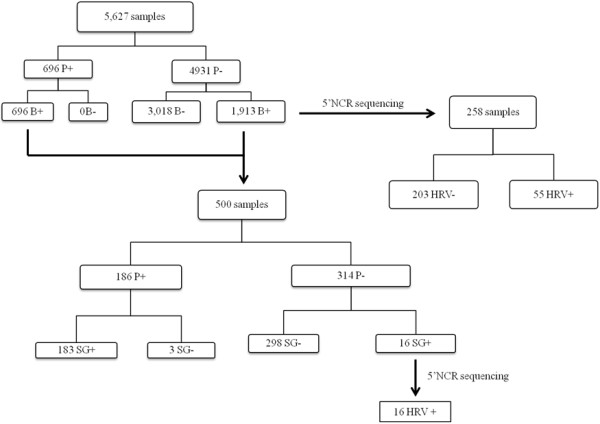
**Evaluation of molecular diagnostic systems.** P: Probe; B: BOXTO; SG: SYBR Green.

– The Taqman probe identified 696 out of 5,627 samples (12,4%) as HRV positive ([P+] samples).

– The BOXTO intercalating agent detected all of the 696 [P+] samples with a specific melting curve peak between 84 and 86.5°C ([P + B+] samples). However, 1,913 out of the 4,931 [P-] samples also provided positive BOXTO results (total [B+]: 2,609 (46.3%)). A random sample of 258 PCR products from these [P-B+] samples was submitted to sequencing: only 24.3% (55/258) contained HRV sequences. Results are summarised in Figure 1.

ii) SYBR Green assay:

In a random selection of 500 [B+] samples tested in parallel with the Taqman probe and the SYBR green assays, 183 (36.6%) were positive in both tests ([P + SG+] samples), 298 were [P-SG-], 3 were [P + SG-] and 16 [P-SG+]. SYBR green positive samples were associated with a melting curve peak between 81 and 84°C.

Ion Torrent sequencing of PCR products of the 16 [P-SG+] samples allowed identification of HRV sequences in all cases (Figure 1).

In the absence of reference test usable as a “gold standard” for the detection of HRV, it is difficult to provide a simple robust analysis of the performances of the different tests. In particular, there is most probably a proportion of our HRV-negative samples that would be identified as HRV-positive using other detection assays (*e.g.*, other primers [46]) and therefore we are not able to provide a strict numbering of true and false negatives for our tests.

Here, we have used a few simple rules to compare the different assays and provide estimates of their performances:

(i) We have considered that [P+] samples were true positives, based on the considerable information available from the literature that suggests that probe-based real-time PCR systems are associated with a negligible rate of false positive results.

(ii) We have considered that the sequencing of amplicons was the reference test for determining if PCR products detected by the BOXTO and SYBR green intercalating agents resulted from the specific amplification of HRV genomes.

Accordingly, when the Taqman probe assay was compared with the SYBR Green assay, a total of 202 “true positive” samples was detected: 186 (92.1%) by the probe assay and 199 (94.9%) by the SYBR Green assay and confirmed by sequencing). Whatever the definition of “negatives”, this means that the sensitivity of the SYBR Green assay is better than that of the probe-based assay. As sequencing of SYBR Green amplicons did not identify any false positive, this also means that the specificity and positive predictive value of the SYBR Green assay are 100% in the sample investigated.

When the Taqman probe assay was compared with the BOXTO assay, 696 “true positive” samples were detected by the probe assay but a number of additional positives were detected by the BOXTO assay (of which an estimate of 24.3% were true positives, based on sequencing experiments). Whatever the definition of “negatives”, this means that the sensitivity of the BOXTO assay is better than that of the probe-based assay. However, sequencing of BOXTO amplicons from [P-B+] samples provided paradoxical results:

– it evidenced a large proportion (~75%) of false positives, which means that the specificity and positive predictive value of the BOXTO assay would be very low in the sample investigated (estimates are <70% and <50%, respectively, hypothesising the absence of false negative results).

– the proportion of confirmed positives (~25%) indicates that a significant proportion of HRV positive samples are not detected by the probe-based assay.

It was interesting to analyse the sequence corresponding to the probe in amplicons from [P-B+] and [P-SG+] samples. Interestingly, the alignment of 11 HRV sequences from [P-B+] samples showed only 3 single substitutions located in the region targeted by the probe, at position 8 (C → T), 15 (T → C), and 20 (C → T) respectively, whilst the remaining 8 sequences were identical to the expected sequence.

Similarly, alignment of 16 [P-SG+] sequences identified only one modified sequence in the region targeted by the probe (position 8, C → T), the remaining 11 sequences being identical to the expected sequence.

**Figure 2:**
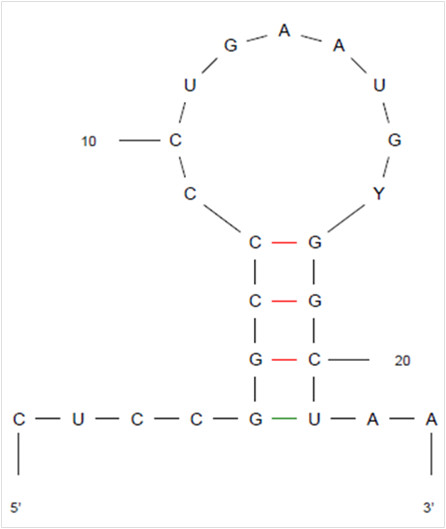
Predicted secondary structure of probe sequence, using the M-fold v3.2 on-line software.

This suggested that different mechanisms could contribute to the inefficient probe-based detection of some HRV sequences. Besides the plausible role of specific mutations in the region targeted by the probe, other mechanisms, obviously independent from this sequence occurred. Using the on-line software M-fold v3.2, the secondary structure of the Taqman probe sequence was determined and showed a “loop-step” conformation (Figure 2), which may hamper the hybridisation process.

## Discussion

Despite the importance of ARIs in terms of morbidity as well as infant mortality, microbiological aetiologies of acute respiratory infections represent a complex, which has yet to be fully characterized in developed countries and remains largely unknown in developing countries. Dual viral infections are common, and a large proportion of children have evidence of viral-bacterial co-infection. In adults, viruses are the putative causative agents in a third of cases of community-acquired pneumonia, in particular influenza viruses, rhinoviruses, and coronaviruses [50]. Accordingly, much better characterization of the epidemiology of ARIs is still needed to improve understanding of the role of the different pathogens and of bacterial-viral interaction in the pathogenesis of ARIs. Since human rhinoviruses represent the most frequent cause of ARIs worldwide and in all age groups, improving the biological diagnosis of rhinoviral infections is a crucial issue.

In this study, we have tested and compared three different HRV molecular diagnostic assays. First, it is obvious that a proportion of samples truly infected by HRVs could not be detected using the Taqman probe test. This is evidenced by a number of probe-negative/BOXTO positive and probe-negative/SYBR green positive samples for which the presence of HRV amplicons was confirmed by direct sequencing of the PCR products. This phenomenon was shown to be highly reproducible and the same HRV positive samples were repeatedly detectable or undetectable using the probe-based assay (data not shown). Since all assays used the same amplification primers, the most likely explanation is a default in probe hybridisation. Analysis of the genomic sequence of HRVs that could not be detected by the probe-based assay showed that only a limited proportion of strains had mutations in the probe hybridisation region. Other mechanism(s) must therefore take place to explain the default of hybridisation of the probe. A possible explanation may lie in the highly structured form of the rhinoviral 5”NCR, which contains an Internal Ribosome Entry Site (IRES). On the one hand, the probe hybridisation region itself is located in a stem-loop region, and on the other hand mutations in different parts of the NCR may modify the secondary structure of the genomic RNA molecule and possibly limit the access to the probe hybridisation region, as previously reported [51]. This is, to our knowledge a rare example of inefficient probe-based detection that is not related to mutations in the probe hybridisation genomic region.

Second, as a number of HRV positive samples that could not be detected using a probe-based assay were identified by DNA-dye-complexes (using BOXTO or SYBR green molecules), it is interesting to consider the performances of these assays. Ideally, the probe-based detection and the intercalating agent-based reaction should be performed in the same reaction tube and detected in distinct reading canals. This is what was attempted by combining a FAM-TAMRA labelled probe and BOXTO. This system allowed the detection of a proportion of probe-negative samples, but the proportion of false positive results was so high that the use of this assay is not possible for clinical or epidemiological purposes (unacceptably low estimated specificity and positive predictive values). It remains from this experiment the important information that a significant proportion of Taqman-negative samples provided true positive results using the same primers and the BOXTO intercalating agent. Accordingly, we tested a number of BOXTO-positive samples using a standard Taqman assay and (separately) a standard SYBR green assay. The SYBR green assay was able to provide positive results in probe-negative samples, which, contrary to what was observed with BOXTO, were all confirmed by direct sequencing of amplicons, indicating that the SYBR green assay is associated with high specificity and positive predictive values. However, the assay failed to detect a few probe-positive samples. Finally, we investigated a new experimental procedure by combining the SYBR Green and a Texas-Red labelled probe. Preliminary experiments on 30 respiratory samples showed that the level of interference between Texas red-based and SYBR Green-based detections in a combined assay was lower than previously observed in the case of the couple (FAM-Boxto), but still an obstacle to routine use: *(i)* in 13samples testing negative using the probe or the SYBR Green alone, a combination of Probe + SG provided 11 P-SG- samples and 2 P + SG- samples (*e.g.*, 2 false positives in 13 negative samples tested, which is too high for diagnostic use, but much lower than with the couple FAM-Boxto (*circa* 75%)); *(ii)* in 17 samples testing positive using the probe or the SG alone, the combination of Probe + SG provided 16 P + SG + samples and one P-SG + sample. The mean detection CT was slightly increased when detection was performed in the presence of SYBR Green and this was sufficient to make negative using the combined assay one sample that tested positive using the probe alone.

This final experiment reinforces the idea that it should be possible to decrease the level of interference between the probe-based and intercalating agent-based detection systems and the next steps should include a systematic investigation of the possible combinations that may allow to identify a couple usable for diagnostic purposes.

## Conclusion

In conclusion, this study confirms the difficulty in detecting HRVs in clinical samples using a single molecular detection system. Besides the possible utilisation of distinct pairs of primers to adequately cover the natural genetic diversity of HRV isolates [46] our results suggest that the optimisation of the detection of amplicons produced by primers is an important issue. In our hands, a SYBR green-based assay was slightly more sensitive that a Taqman probe-based assay and constituted the best compromise amongst the techniques tested. Attempts to associate a probe-based detection with an intercalating agent-based detection should be renewed to identify a reliable couple (DNA dye/probe label) that could be used for routine diagnostic and epidemiological detection of human rhinoviruses.

## Competing interests

The authors declare that they have no competing interest.

## Authors’ contributions

JD performed the experiments, analysed the data and wrote the manuscript. LN conceived and designed the experiments, analysed the data and wrote the manuscript. VF performed the experiments, analysed the data and wrote the manuscript. OP performed the experiments and analysed the data. CG and LTP designed the experiments. XdL conceived and designed the experiments, analysed the data, wrote the manuscript and supervised the work. All authors read and approved the final manuscript.
